# Supplementary material for: USP37 prevents premature disassembly of stressed replisomes by TRAIP
Source: Nat Commun. 2025 Jun 18;16:5333. doi: 10.1038/s41467-025-60139-z (PMC12177040; doi:10.1038/s41467-025-60139-z)
Supplement: Supplementary file 1 — Supplementary Information [file 41467_2025_60139_MOESM1_ESM.pdf]

Supplementary Information

USP37 prevents premature disassembly of stressed replisomes by TRAIP

Olga V. Kochenova[1,2]*, Giuseppina D'Alessandro[3,4]*, Domenic Pilger[5†], Ernst Schmid[1†], Sean L. Richards[3], Marcos Rios Garcia[6], Satpal S. Jhujh[6], Andrea Voigt[3], Vipul Gupta[3], Christopher J. Carnie[3], R. Alex Wu[1], Nadia Gueorguieva[3], Simon Lam[3], Grant S. Stewart[6], Johannes C. Walter[1,2#] and Stephen P. Jackson[3#]

Affiliations:
[1] Department of Biological Chemistry and Molecular Pharmacology, Harvard Medical School, Blavatnik Institute; Boston, MA 02115, USA
[2] Howard Hughes Medical Institute; Boston, MA 02115, USA.
[3] Cancer Research UK Cambridge Institute, Li Ka Shing Building, Robinson Way, Cambridge CB2 0RE, UK
[4] Present address: IFOM ETS, The AIRC Institute of Molecular Oncology, Milan, Italy
[5] The Gurdon Institute and Department of Biochemistry, University of Cambridge
[6] Institute of Cancer and Genomic Sciences, College of Medical and Dental Sciences, University of Birmingham, Birmingham, UK

[*,†] Equal contribution

[#] Corresponding authors. Emails: steve.jackson@cruk.cam.ac.uk, johannes_walter@hms.harvard.edu

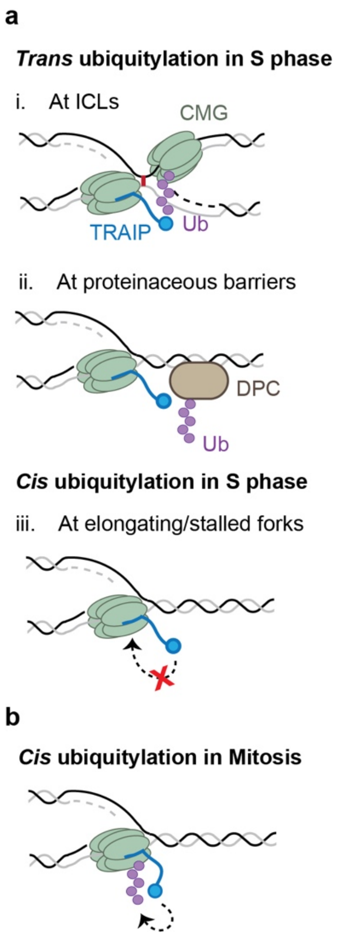

Supplementary Fig. 1: Model for TRAIP function in S and M phases.
a, Using its *trans*-ubiquitylation mode in S phase, TRAIP can ubiquitylate CMGs that have converged on an ICL (i) or DPCs and other obstacles encountered by the replisome (ii), but not the replisome it travels with (which we call *cis* ubiquitylation) (iii). **b,** In mitosis, TRAIP undergoes a conformational change that allows it to *cis* ubiquitylate the CMG it travels with.

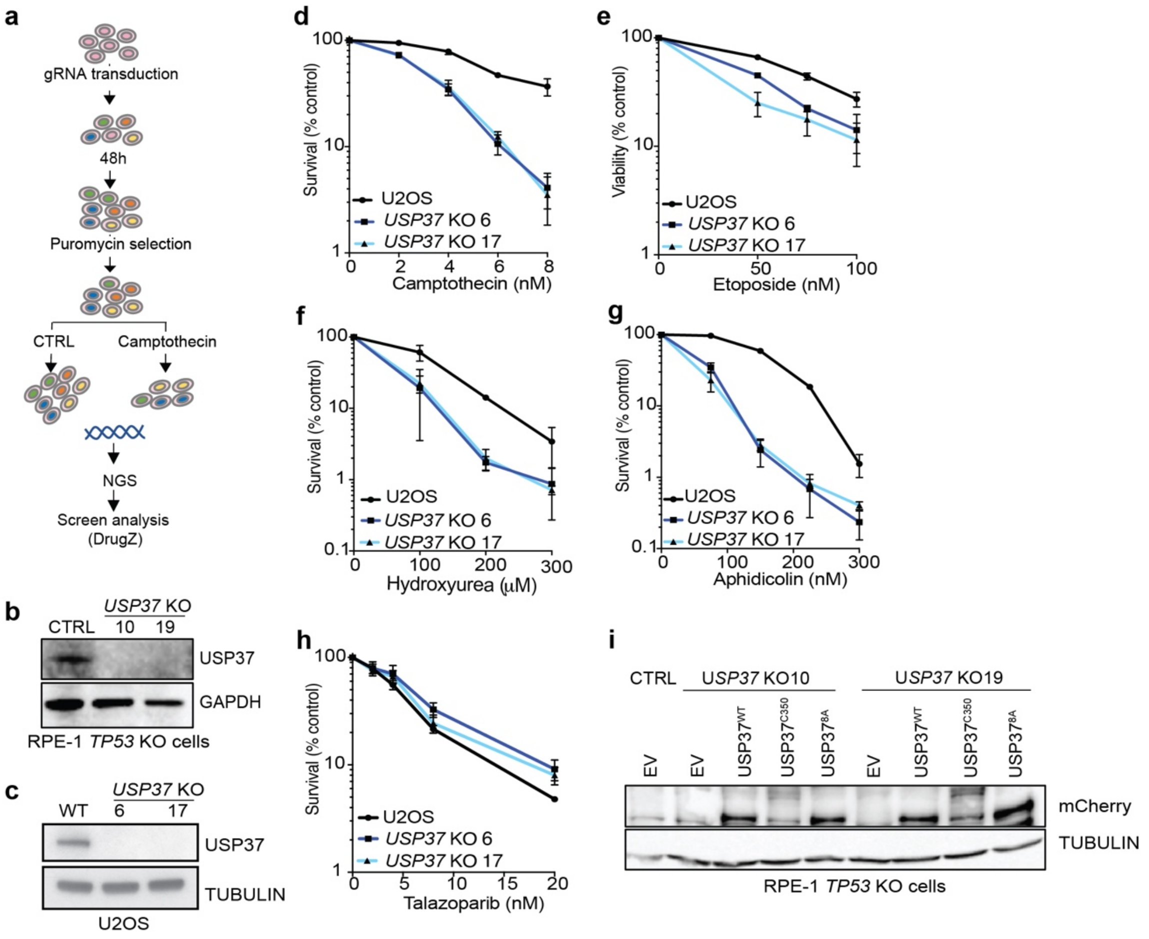

Supplementary Fig. 2: *USP37* knock-out hypersensitises to topoisomerase inhibitors and replication stress inducing agents.

a, Schematic of the CRISPR screen in U2OS cells. **b-c,** Western blot validation of *USP37* KO in RPE-1 *TP53* KO cells (**b**) and U2OS cells (**c**). **d-h,** Clonogenic survival assays of WT and *USP37* KO U2OS cells upon treatment with (**d**) camptothecin, (**e**) etoposide, (**f**) hydroxyurea, (**g**) aphidicolin or (**h**) talazoparib. n=2 independent experiments with consistent results, with the exception of d and h where n=3 independent experiments. Bars represent means ± SEM. **i,** Western blot validation of mCherry-USP37^{WT}, mCherry-USP37^{C350A} (catalytically inactive), or mCherry-USP37^{8A} expression in *USP37* knockout cells. Source data are provided as a Source Data file.

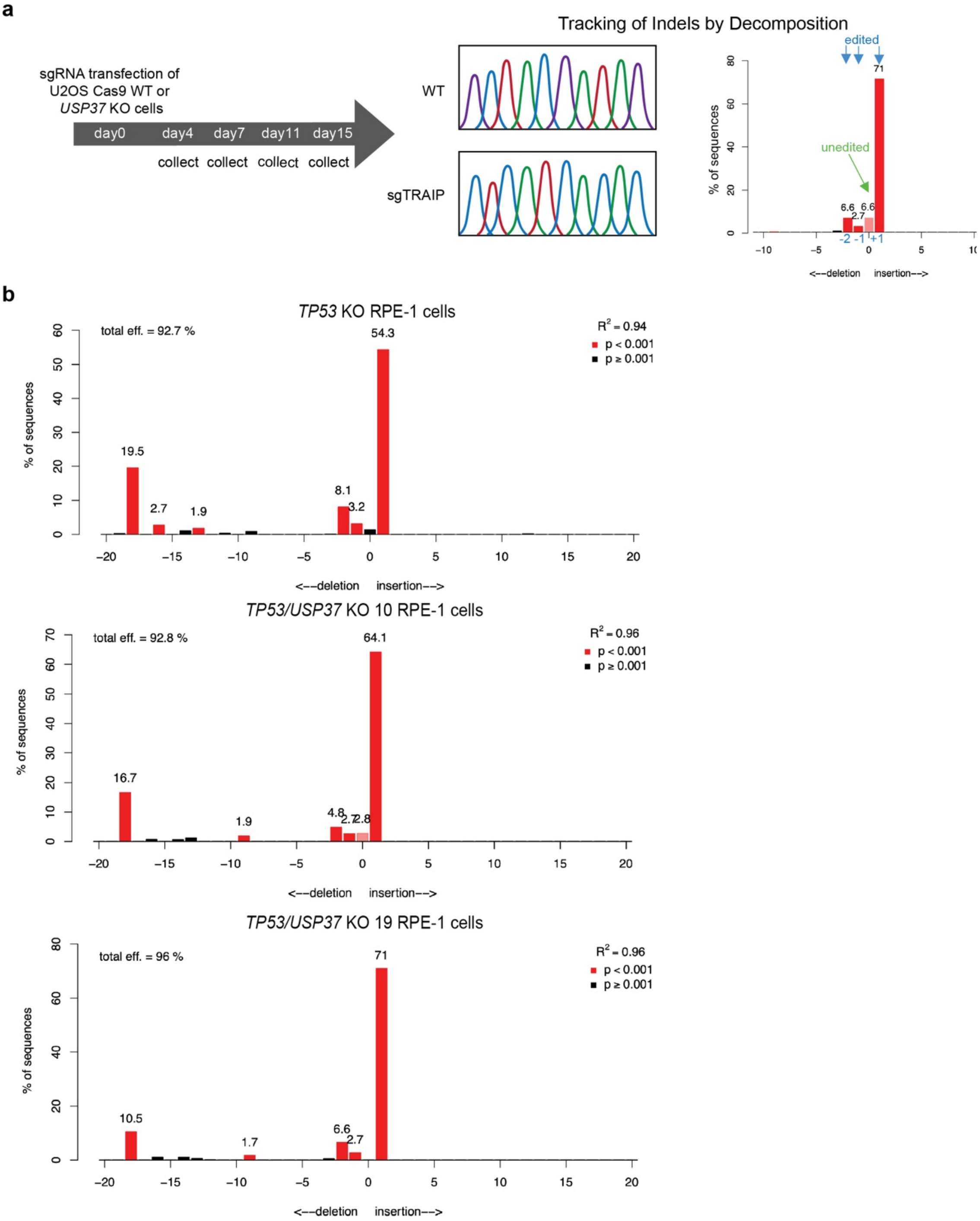

Supplementary Fig. 3: Validation of *TRAIP* KO in *TP53* KO and *TP53/USP37* KO RPE-1 cells.

a, Scheme of the tracking indels by decomposition (TIDE)-based cell competition assay shown in Fig. 2b. Scheme generated with BioRender. **b,** TIDE-validatio of *TRAIP* KO in *TP53* KO and *TP53/USP37* KO RPE-1 cells.

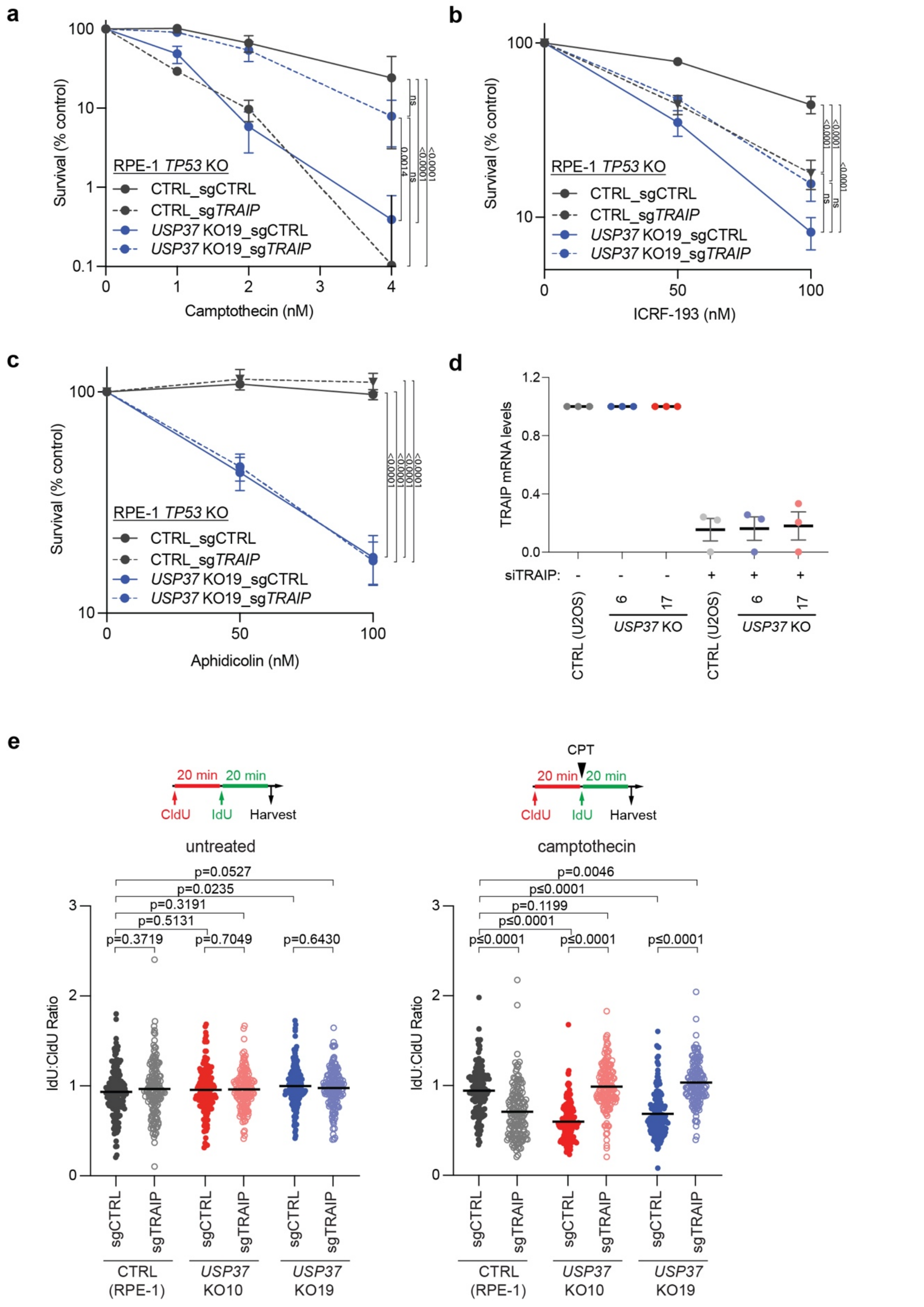

Supplementary Fig. 4: TRAIP loss improves the viability of *USP37* knockout cells upon treatment with topoisomerase inhibitors but not aphidicolin.

a-c, Clonogenic survival assays as in Fig. 2c-e of CTRL or a different *USP37* knockout (KO) clone transduced with a control sgRNA (LacZ) or with a sgRNA targeting *TRAIP* upon treatment with camptothecin (**a**), ICRF-193

(**b**) or aphidicolin (**c**). The CTRL data are the same as in Fig. 2c-e. n=3 independent experiments. Bars represent means ± SEM. Half plot points in **c** indicate zero percent viability. Statistical analysis was performed using two-way ANOVA with Tukey's test for multiple comparisons. **d,** RT-qPCR experiment to monitor TRAIP knock-down efficiency. n=2 independent experiments. Bars represent means. **e,** Top, schematics depicting experimental design. Cells were incubated with CldU for 20 min, and then pulse-labeled with IdU for 20 min in the presence or absence of camptothecin (CPT). Bottom, comparative ratios of IdU:CldU track length in WT or *USP37* knockout (KO) clones transduced with a control sgRNA or with a sgRNA targeting *TRAIP* in untreated (left) conditions or upon treatment with camptothecin (right). The black line represents the mean. Untreated: n=146 (CTRL, sgCTRL), 154 (CTRL, sgTRAIP), 150 (*USP37* KO10, sgCTRL), 150 (*USP37* KO10, sgTRAIP), 145 (*USP37* KO19, sgCTRL), 160 (*USP37* KO19, sgTRAIP) cells; camptothecin: n=132 (CTRL, sgCTRL), 157 (CTRL, sgTRAIP), 149 (*USP37* KO10, sgCTRL), 159 (*USP37* KO10, sgTRAIP), 156 (*USP37* KO19, sgCTRL), 153 (*USP37* KO19, sgTRAIP) cells. Cells were analyzed from 3 biological replicates. Statistical analysis was performed using unpaired two-tailed Mann-Whitney test. Source data are provided as a Source Data file.

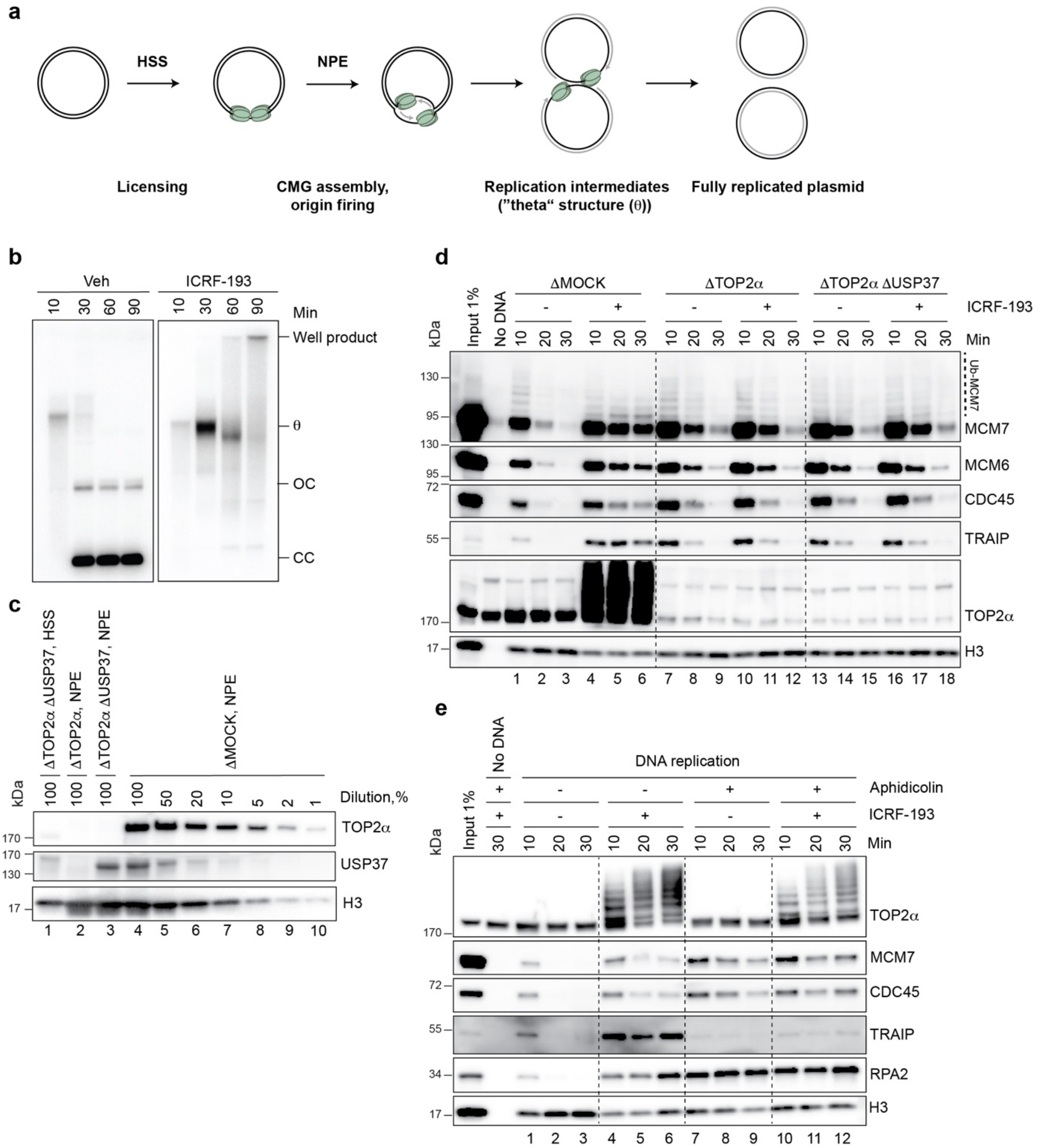

Supplementary Fig. 5: CMG stalling during topological stress requires trapping of TOP2α on DNA.

a, A schematic depicting plasmid replication in *Xenopus* egg extracts. HSS, high speed supernatant, is used for replication licensing. Subsequently, NPE, nucleoplasmic extract, is used to initiate replication from licensed plasmids. **b,** Plasmid DNA was replicated in the presence or absence of 200 μM ICRF-193 in extracts containing $[\alpha\text{-}^{32}P]$dATP. Replication intermediates were then separated on a native agarose gel and visualized by autoradiography. At early time points, replication of plasmid DNA generates a "theta" structure (θ) (see Supplementary Fig. 5a), a late replication intermediate formed when replisomes converge, that is subsequently converted to fully replicated closed and open circular products ("CC" and "OC", respectively). ICRF-193 delays conversion of theta structures to CC and OC, indicative of impaired replisome convergence, and causes accumulation of a smear that probably represents highly catenated daughter molecules (Ref.[1], see Supplementary Fig. 2a). Samples are from the same experiment; the images are separate agarose gels, which were processed and imaged in parallel. **c,** Western blot analysis of mock, USP37, and TOP2α depletions. Related to Supplementary Fig. 5d. **d,** Plasmid DNA was incubated in the indicated egg extracts in the presence or absence

of 200 μM ICRF-193. At the specified times, chromatin was recovered and blotted for the indicated proteins. **e,** Plasmid DNA was incubated in the indicated egg extracts in the presence or absence of 200 μM ICRF-193 and 50 ng/μl aphidicolin. At specified times, chromatin was recovered and immunoblotted for the indicated proteins. Aphidicolin inhibits DNA synthesis and thus prevents formation of pre-catenanes while still allowing DNA unwinding and accumulation of supercoils[2–4], which are presumably located ahead of the fork. As expected, aphidicolin increased RPA binding and decreased TOP2α recruitment to chromatin (lane 7 vs 1)[1]. However, in the presence of aphidicolin, ICRF-193 still caused accumulation of TOP2α on DNA, albeit to a lesser extent (lanes 10-12 vs 4-6) suggesting that TOP2α can be trapped on supercoils ahead of the replisome. Source data are provided as a Source Data file.

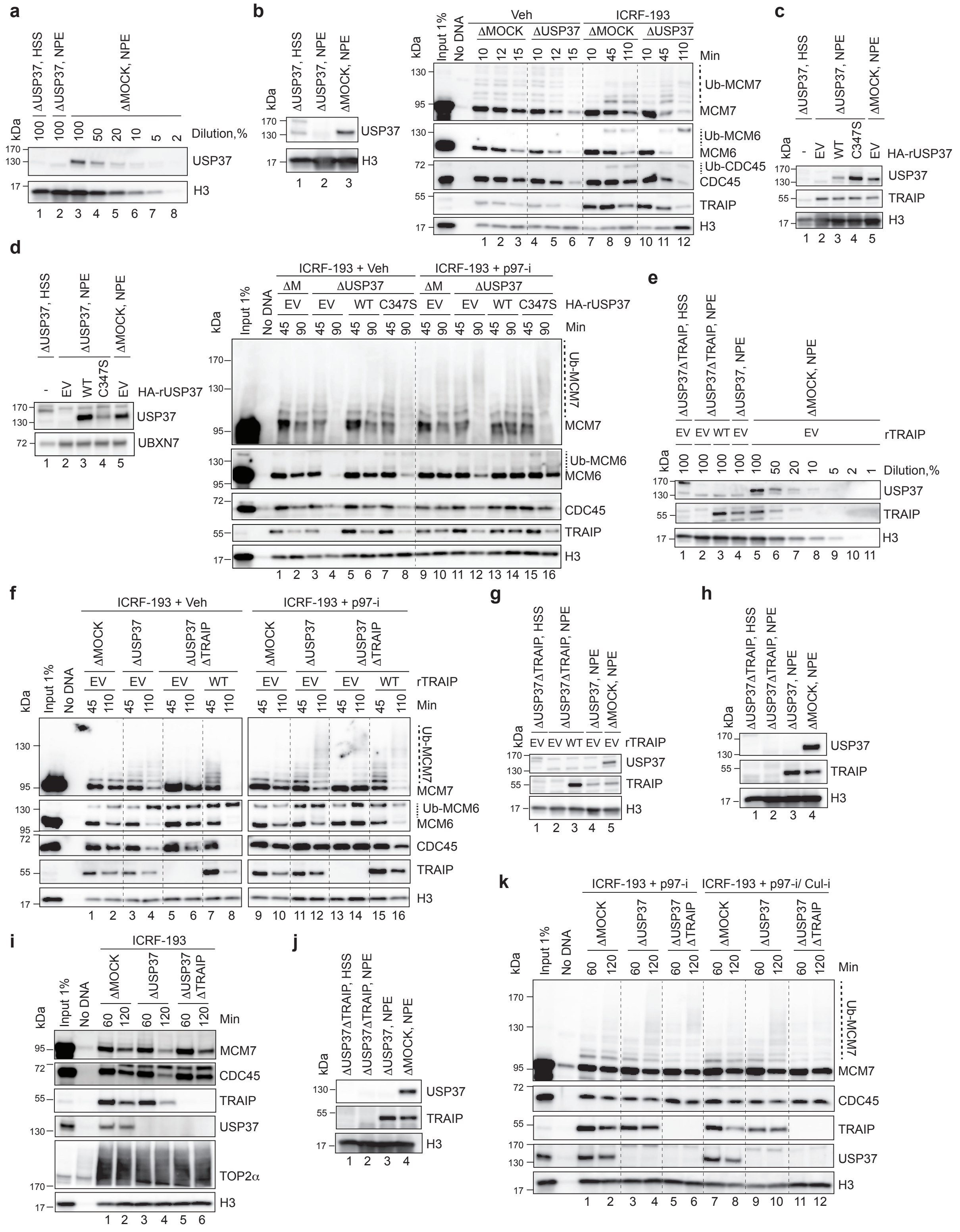

Supplementary Fig. 6: USP37 prevents premature CMG unloading by TRAIP during topological stress.

a, Western blot analysis of mock and USP37 depletions. **b**, An independent replicate of Fig. 3a. Left, Western blot analysis of mock and USP37 depletions. Right, plasmid pull-down assay. See Fig. 3a for more details. **c,** Western blot analysis of mock and USP37 depletions supplemented with recombinant USP37 expressed in wheat germ extract. Abbreviations as in Fig. 3b. **d**, An independent replicate of Fig. 3b. Left, Western blot analysis of mock and USP37 depletions supplemented with recombinant USP37 expressed in wheat germ extract. Right, plasmid pull-down assay. See Fig. 3b for more details. Note that apparent underloading of $USP37^{C347S}$ is probably

due to this protein's hyperubiquitylation. **e,** Western blot analysis of mock, USP37, and TRAIP depletions supplemented with recombinant TRAIP expressed in wheat germ extract. Abbreviations as in Fig. 3c. **f**, An independent replicate of Fig. 3c. Samples are from the same experiment; blots were processed in parallel. **g,** Western blot analysis of mock, USP37, and TRAIP depletions supplemented with recombinant TRAIP expressed in wheat germ extract. Related to Supplementary ig. 6f. Abbreviations as in Fig. 3c. **h,** Western blot analysis of mock and USP37 depletions. Related to Supplementary Fig. 6i. **i,** *Xenopus* sperm chromatin was replicated in the indicated extracts in the presence of of 200 μM ICRF-193. At the specified times, chromatin was recovered and immunoblotted for the indicated proteins, which demonstrated enhanced CMG unloading in the absence of USP37 that depended on TRAIP (compare lanes 2, 4, 6). **j,** Western blot analysis of mock and USP37 depletions. Related to Supplementary Fig. 6k. **k,** Same as Supplementary Fig. 6i, but 55 minutes after replication initiation, replication reactions were supplemented with 200 μM NMS-873 (p97-i) or 200 μM MLN4924 (Cul-i), as indicated. Since there are more termination events on sperm chromatin, Cul-i was added in some reactions to reduce the background $CRL2^{LRR1}$-specific ubiquitylation of MCM7, which revealed enhanced TRAIP-dependent CMG ubiquitylation in USP37-depleted extracts (compare lanes 8, 10, 12). Source data are provided as a Source Data file.

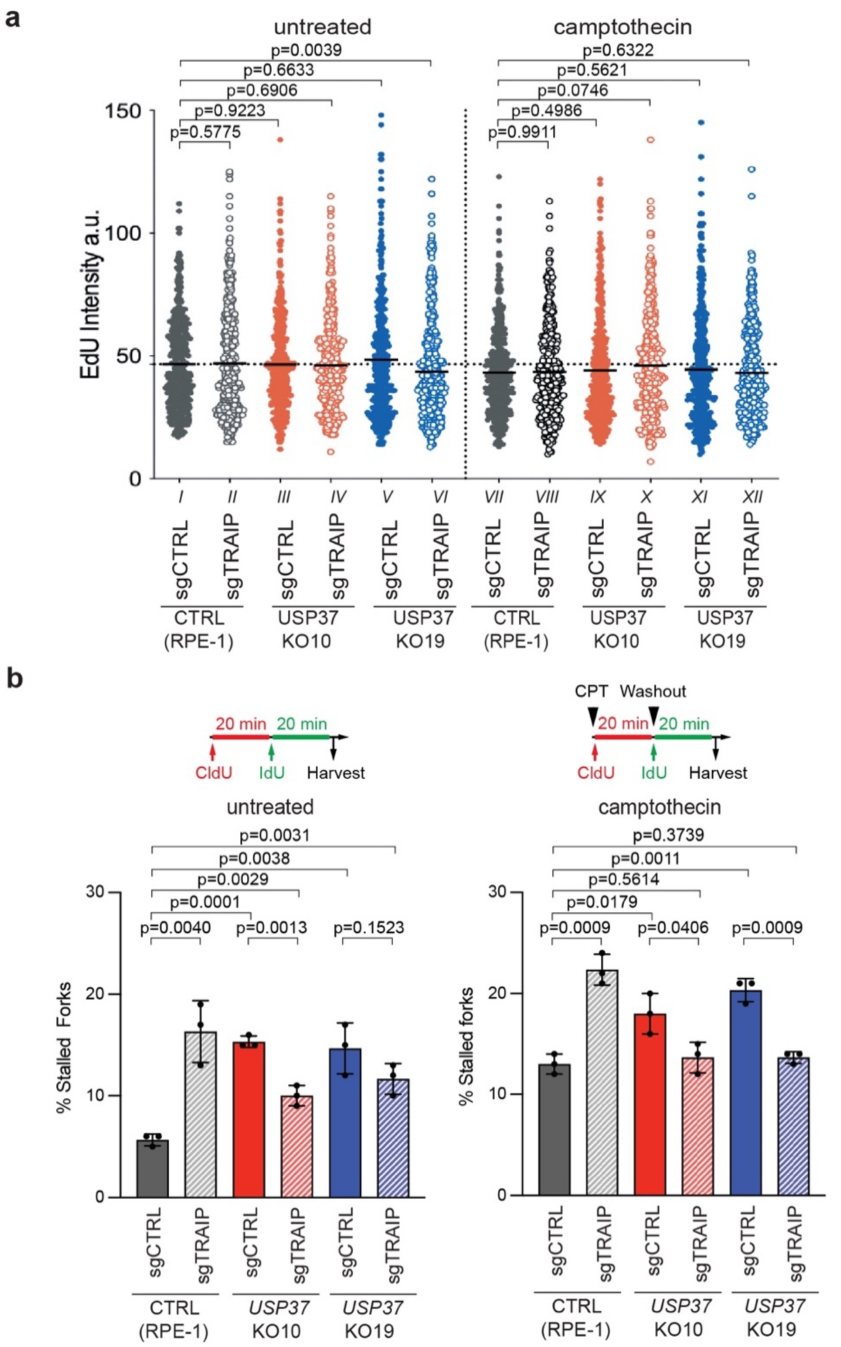

Supplementary Fig. 7: TRAIP loss promotes fork restart in *USP37* knockout cells following the removal of camptothecin.

a, Dot plot indicating the intensity of EdU (indicating replicating DNA) in untreated or camptothecin treated RPE-1 *TP53* KO CTRL or *USP37* KO cells. The black line represents the mean EdU intensity. Untreated: n=446 (CTRL, sgCTRL), 392 (CTRL, sgTRAIP), 459 (*USP37* KO10, sgCTRL), 371 (*USP37* KO10, sgTRAIP), 387 (*USP37* KO19, sgCTRL), 392 (*USP37* KO19, sgTRAIP) cells; camptothecin: n=450 (CTRL, sgCTRL), 363 (CTRL, sgTRAIP), 453 (*USP37* KO10, sgCTRL), 412 (*USP37* KO10, sgTRAIP), 375 (*USP37* KO19, sgCTRL), 381 (*USP37* KO19, sgTRAIP) cells. Cells were analyzed from 3 biological replicates. Statistical analysis was performed using unpaired two-tailed Mann-Whitney test. **b,** Analysis of replication fork restart following camptothecin treatment. RPE-1 *TP53* KO CTRL or *USP37* KO cells were labelled and treated as indicated in the experimental scheme to estimate the number of restarting forks in untreated conditions (left) and after camptothecin treatment (right). The graphs show the percentage of stalled forks calculated from the total number of CldU positive forks. Bar represents mean ± SEM. n=3 independent experiments. Statistical analysis was performed using unpaired two-tailed Student's t test. Source data are provided as a Source Data file.

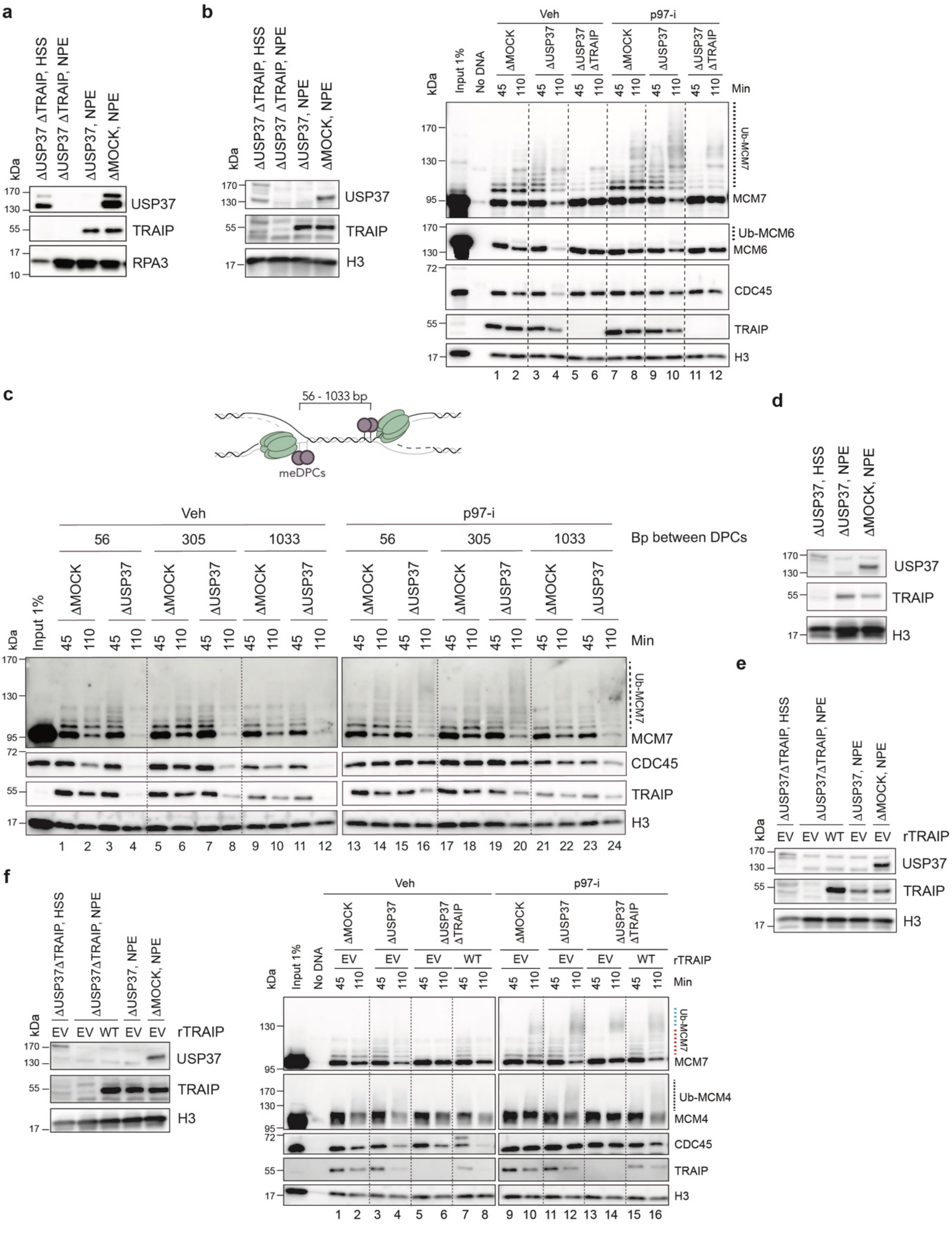

Supplementary Fig. 8: TRAIP promotes premature disassembly of CMGs stalled at distances of up to 1 kb.

a, Western blot analysis of mock, USP37, and TRAIP depletions. **b,** An independent replicate of Fig. 4a. Right, Western blot analysis of mock, USP37, and TRAIP depletions. Left, plasmid pull-down assay. **c,** Top, schematic of the meDPC substrates used, including the variable distance between distal leading strand meDPCs. The indicated meDPC substrates were incubated in egg extracts in the presence or absence of 200 μM p97-i. At the specified times, chromatin was recovered and blotted for the indicated proteins. USP37 depletion efficiency is shown in Supplementary Fig. 8d. Samples are from the same experiment; blots were processed in parallel. **d,**

Western blot analysis of mock and USP37 depletions. **e,** Western blot analysis of mock, USP37, and TRAIP depletions supplemented with recombinant TRAIP expressed in wheat germ extract. Abbreviations as in Fig. 4b. **f,** An independent replicate of Fig. 4b. Right, Western blot analysis of mock, USP37, and TRAIP depletions supplemented with recombinant TRAIP expressed in wheat germ extract. Left, plasmid pull-down assay. Abbreviations as in Fig. 4b. Samples are from the same experiment; blots were processed in parallel. Source data are provided as a Source Data file.

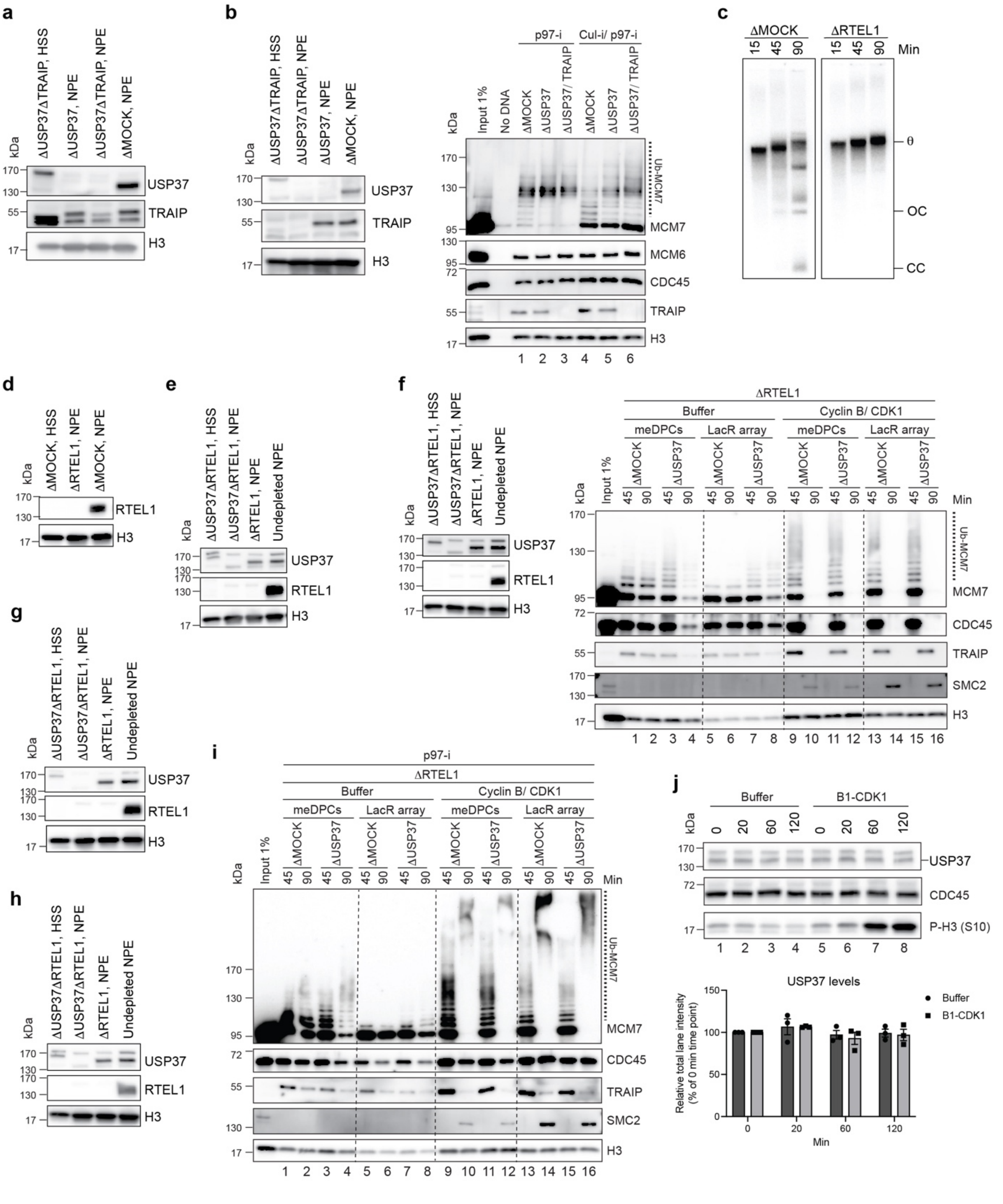

Supplementary Fig. 9: TRAIP ubiquitylates stalled CMGs *in trans*.

a, Western blot analysis of mock, USP37, and TRAIP depletions. **b,** An independent replicate of Fig. 4c. Right, Western blot analysis of mock, USP37, and TRAIP depletions. Left, plasmid pull-down assay. **c,** Plasmid DNA was pre-incubated with LacR and then replicated in the presence or absence of RTEL1 in extracts containing [α-^{32}P]dATP. Replication intermediates were separated on a native agarose gel and visualized by autoradiography. RTEL1 depletion delayes progression of replication forks through the LacR array, as seen by stabilization of the theta structure (θ) and absence of fully replicated closed and open circular products ("CC" and "OC", respectively). Samples are from the same experiment; the images are part of the same agarose gel, which was cropped to remove irrelevant information. RTEL1 depletion efficiency is shown in Supplementary Fig. 9d. **d,** Western blot analysis of mock and RTEL1 depletions. **e,** Western blot analysis of USP37 and RTEL1 depletions. **f,** An independent replicate of Fig. 4d. Right, Western blot analysis of mock, USP37, and TRAIP depletions. Left, plasmid pull-down assay **g,** Western blot analysis of USP37 and RTEL1 depletions. **h**, Western blot analysis of

USP37 and RTEL1 depletions. Related to Supplementary Fig. 9i. **i**, An independent replicate of Fig. 4e. **j,** Top, representative western blot images of total USP37 levels during replication in interphase (Buffer) or mitotic (B1-CDK1) egg extracts. P-H3, phosphorylated H3 at S10 residue. Bottom, quantification of total USP37 amount in replication reactions normalized to CDC45. n=3 independent experiments. Bar represents mean + SEM. Source data are provided as a Source Data file.

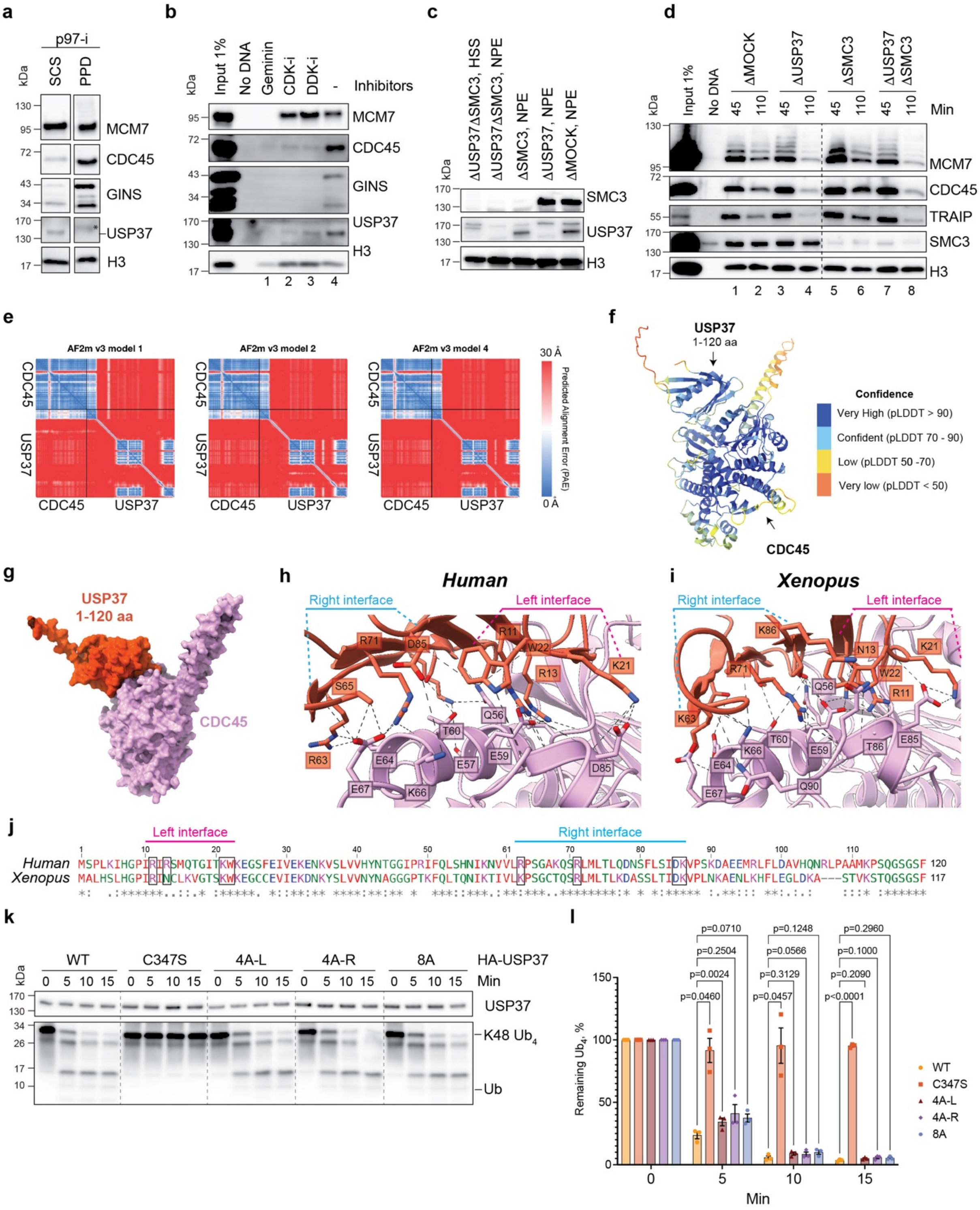

Supplementary Fig. 10: USP37's PH domain is predicted to interact with CDC45.

a, Side-by side comparison of USP37 recovery in sperm chromatin spindown (SCS) and plasmid pulldown (PPD) procedures. *Xenopus* sperm chromatin or plasmid DNA were incubated in egg extracts supplemented with indicated inhibitors for 15 min, recovered and immunoblotted. The images are all part of the same Western blot, which was cropped to remove irrelevant information. Black asterisk, a smudge that is not a USP37 band. **b,** An independent replicate of Fig. 5a. **c,** Western blot analysis of mock-, USP37-, SMC3-, and USP37/SMC3-depletions. Related to Supplementary Fig. 10d. **d,** The meDPCs substrate (pDPC4-1033) was incubated in indicated egg extracts, recovered and immunoblotted. Depletion efficiencies are shown in Supplementary Fig. 10c. **e,** Predicted alignment error (PAE) plots generated by the 3 AF-M models for the complex of human USP37 and CDC45. **f,** AlphaFold-Multimer (AF-M) prediction of human CDC45 and USP37 interaction colored by pLDDT value, a measure of the confidence of local amino acid positioning. For simplicity, only amino acid

residues (aa) 1-120 corresponding to the USP37 PH domain are shown. **g,** Space filling representation of the same model shown in Supplementary Fig. 10f colored by chain. **h,** Close-up views of key *Human* USP37 and CDC45 residues that were predicted to interact by AF-M. **i,** Close-up views of key *Xenopus* USP37 and CDC45 residues predicted to interact. **j,** Alignment of human (residues 1-120) and frog (residues 1-117) USP37. Black boxes indicate residues that were substituted to alanines in the $USP37^{8A}$ mutant. "*", conserved residues; ":", conservative substitution; ".", semi-conservative substitution; " ", non-conservative substitution; "-", gap. **k,** A representative *in vitro* deubiquitylation assay with recombinant *Xenopus* HA-USP37 variants expressed in wheat germ extract. HA-USP37 was immobilized on anti-HA magnetic beads and incubated with K48-linked tetraubiquitin (Ub_4). **4A-L**, R11A, N13A, K21A, W22A; **4A-R**, K63A, R71A, D85A, K86A; **8A**, R11A, N13A, K21A, W22A, K63A, R71A, D85A, K86A. **l,** Quantification of the remaining K48-linked tetraubiquitin (Ub_4) in Supplementary Fig.10 normalized to USP37 levels. n=3 independent experiments. Bars represent means ± SEM. Statistical analysis was performed using two-way ANOVA and Dunnett test for multiple comparisons. Source data are provided as a Source Data file.

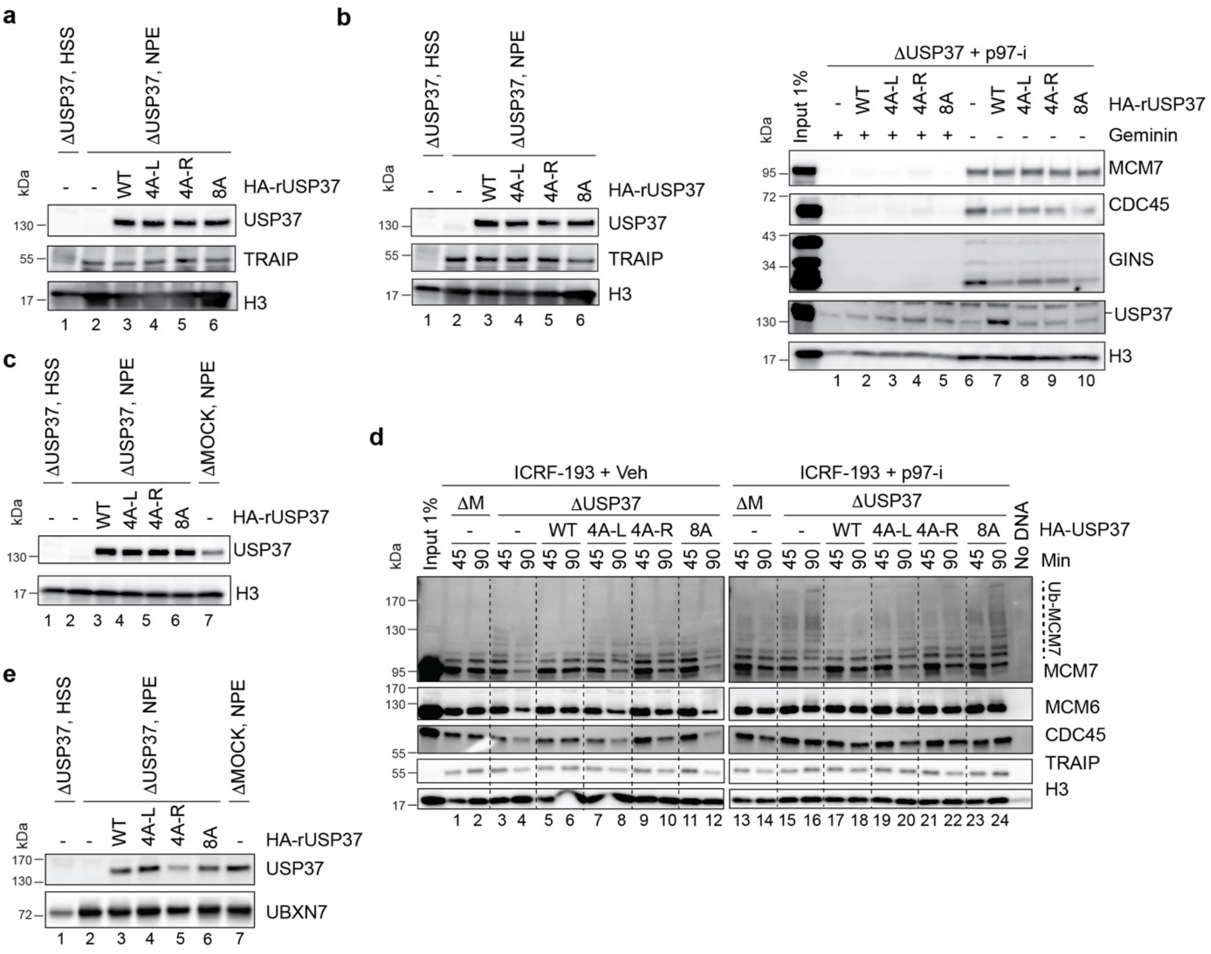

Supplementary Fig. 11: USP37 interaction with CDC45 is required for its protective function towards topoisomerase inhibitors.

a, Western blot analysis of mock and USP37 depletions supplemented with recombinant USP37 expressed in wheat germ extract. Abbreviations as in Fig. 5c. **b,** An independent replicate of Fig. 5c. Left, Western blot analysis of mock and USP37 depletions supplemented with recombinant USP37 expressed in wheat germ extract. Abbreviations as in Fig. 5c. Right, plasmid pull-down assay. **c,** Western blot analysis of mock- and USP37-depletions supplemented with recombinant USP37 expressed in wheat germ extract. **d,** An independent replicate of Fig. 5d. USP37 depletion efficiency and recombinant protein levels are shown in Supplementary Fig. 10e. Samples are from the same experiment; blots were processed in parallel. **e,** Western blot analysis of mock and USP37 depletions supplemented with recombinant USP37 expressed in wheat germ extract. Abbreviations as in Supplementary Fig. 10k. Source data are provided as a Source Data file.

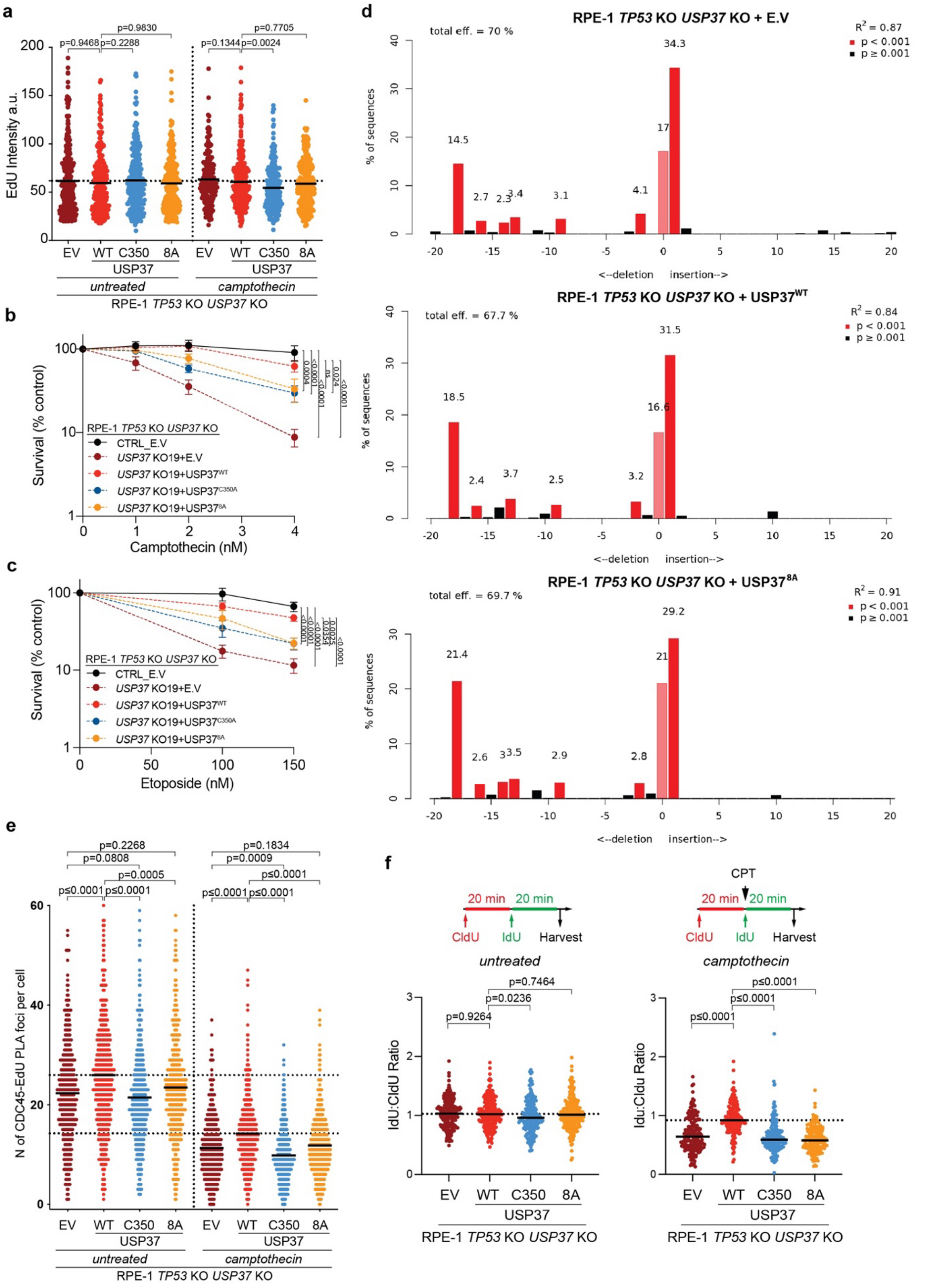

Supplementary Fig. 12: USP37 interaction with CDC45 is required for its protective function towards topoisomerase inhibitors in cells

a, Dot plot indicating the intensity of EdU (indicating replicating DNA) in RPE-1 *TP53* KO *USP37* KO cells expressing mCherry-tagged E.V, USP37^{WT}, USP37^{C350}, or USP37^{8A} untreated or camptothecin treated. The black line represents the mean. Untreated: n=291 (EV), 329 (WT), 307 (C350), 287 (8A) cells; camptothecin: n=197

(EV), 320 (WT), 287 (C350), 300 (8A) cells. Cells were analyzed from 3 biological replicates. Statistical analysis was performed using unpaired two-tailed Mann-Whitney test. **b-c,** Clonogenic survival assays of control (CTRL) cells or *USP37* KO19 cells complemented with vectors expressing mCherry (EV), mCherry-USP37^{WT}, mCherry-USP37^{C350A} (catalytically inactive), or mCherry-USP37^{8A} (defective for CDC45 interaction) upon treatment with camptothecin (**b**) or etoposide (**c**); n=4(b) and 5(c) independent experiments. Bars represent means ± SEM. Statistical analysis was performed using two-way ANOVA with Šidák's test for multiple comparisons. **d**, TIDE-validation of *TRAIP* KO in *TP53 USP37* KO RPE-1 cells complemented with E.V, USP37^{WT}, or USP37^{8A}. **e**, Dot plot indicating the number of PLA foci between CDC45 and EdU (replicating DNA) in *USP37* KO19 cells expressing mCherry (EV), mCherry-USP37^{WT}, mCherry-USP37^{C350A} (catalytically inactive), or mCherry-USP37^{8A} (defective for CDC45 interaction) in untreated conditions (left) or upon treatment with camptothecin (right). The black line represents the mean. Untreated: n=440 (EV), 487 (WT), 424 (C350), 441 (8A) cells; camptothecin: n=389 (EV), 434 (WT), 408 (C350), 405 (8A) cells. Cells were analyzed from 3 biological replicates. Statistical analysis was performed using unpaired two-tailed Mann-Whitney test. **f,** Top, schematics depicting experimental design. Cells were incubated with CldU for 20 min and then pulse-labeled with IdU for 20 min in the presence or absence of camptothecin. Bottom, the comparative ratios of the IdU:CldU track length in *USP37* KO clone cells expressing mCherry (Vector), mCherry-USP37^{WT}, mCherry-USP37^{C350A} (catalytically inactive), or mCherry-USP37^{8A} (defective for CDC45 interaction) in untreated conditions (left) or upon treatment with camptothecin (right). The black line represents the mean IdU:CldU. Untreated: n=239 (EV), 221 (WT), 193(C350), 228 (8A) cells; camptothecin: n=189 (EV), 177 (WT), 186 (C350), 171 (8A) cells. Cells were analyzed from 3 biological replicates. Statistical analysis was performed using unpaired two-tailed Mann-Whitney test. Source data are provided as a Source Data file.

Supplementary Table 1. The sequences and source of oligonucleotides used in the study.

Oligonucleotides	Source	Name
AATGTGGTGCTTCGACCCAG	IDT	USP37 sgRNA
TGGTCTGTAGTCTAGTCATAGCCT	Sigma	US37 TIDE Fw
CCCTTGGTGCAAGATCTCTGT	Sigma	US37 TIDE Rev
GACGTGGCCGCCATCCACTG	IDT	TRAIP sgRNA
TTGCCCAGGCTAACGGTTTT	Sigma	TRAIP TIDE Fw
AGGCGAAGTATTCACGCTCC	Sigma	TRAIP TIDE Rev
CTACATGAACGCCATATTGCAATCTC	IDT	OK74
GAGGTGTTTCCGAGGTTACTGAAG	IDT	OK75
TATCGGACCGTGCAGGGAATG	IDT	OK99
GTACCACTGAACAAAGCGGAGAATTTG	IDT	OK100
ACTCAATCCGGCTCTTCAGCCATATGGT GCACTCTCAGTACAATCTGC	IDT	OK109
ACCACCGCGCAAACGCAG	IDT	OK114
TTGTTAGGGAGGAAACCACCGCG	IDT	OK115
TTGGCTTCAACGTAAACCAC	IDT	OK117
CATTCCCTGCACGGTCCGATAGCCATTG CGTGTCTTAAAGTTGGCACTAGTGCGGC AAAGGAGGGTTGCTGTGAGGTGATAGA GAAAGACAATAAATACTCCCTTGTGGTT AACTATAATGCGGGAGGTGGACCAACA AAATTCCAATTGACACAAAACATTAAGA CAATTGTGCTGGCGCCTAGTGGCTGCAC TCAGTCAGCGTTGATGTTGACTCTGAAG GATGCATCCTCTCTGACTATTGCAGCGG TACCACTGAACAAAGCGGAGAATTTG	IDT	PH-8A gBlock
TCAGCATC[C5-fluor dC]GGTAGCTACTCAATC[C5-fluor dC]GGTACC	IDT	Dual-Top/Top-nt.BbvCI
CAGCATC[C5-fluor dC]GGTAGCTACTCAATC[C5-fluor dC]GGCTCTTCA	IDT	Dual-Top/Top-nt.BspQI
Lincode Non-targeting Control 3	Dharmacon	Cat# D-001810-03
CAG CAU GGU UAC UAC GAA ATT	Sigma[5]	siTRAIP
CGGACCTGTAGCAGTTTCTT	Sigma	TRAIPqPCR Fw
CGAAGAAGTCGGAGCAGATAG	Sigma	TRAIPqPCR Fw

Supplementary References

1. Heintzman, D. R., Campos, L. V, Byl, J. A. W., Osheroff, N. & Dewar, J. M. Topoisomerase II Is Crucial for Fork Convergence during Vertebrate Replication Termination. *Cell Rep* **29**, 422-436 e5 (2019).
2. Michael, W. M., Ott, R., Fanning, E. & Newport, J. Activation of the DNA Replication Checkpoint Through RNA Synthesis by Primase. *Science (1979)* **289**, 2133–2137 (2000).
3. Walter, J. & Newport, J. Initiation of Eukaryotic DNA Replication. *Mol Cell* **5**, 617–627 (2000).
4. Low, E., Chistol, G., Zaher, M. S., Kochenova, O. V & Walter, J. C. The DNA replication fork suppresses CMG unloading from chromatin before termination. *Genes Dev* **34**, 1534–1545 (2020).
5. Feng, W. *et al.* TRAIP regulates replication fork recovery and progression via PCNA. *Cell Discov* **2**, 16016 (2016).
