# Supplementary material for: USP37 prevents premature disassembly of stressed replisomes by TRAIP
Source: Nat Commun. 2025 Jun 18;16:5333. doi: 10.1038/s41467-025-60139-z (PMC12177040; doi:10.1038/s41467-025-60139-z)
Supplement: Supplementary file 2 — Description of Additional Supplementary Files [file 41467_2025_60139_MOESM2_ESM.pdf]

Description of Additional Supplementary Files

File Name: Supplementary Data 1

Description: *Human* and *Xenopus* USP37 and CDC45 pair predictions generated by AF-M. SPOC_score, Structure Prediction and Omics-based Classifier score; avg_models, quantification of the agreement among the three independently trained versions of AF-M; ipTM, the interface predicted Template Modeling score; pDOCKQ, predicted Docking Quotient; PAE, Predicted aligned error; pLDDT, the predicted local distance difference test.
