# Supplementary material for: USP37 prevents premature disassembly of stressed replisomes by TRAIP
Source: Nat Commun. 2025 Jun 18;16:5333. doi: 10.1038/s41467-025-60139-z (PMC12177040; doi:10.1038/s41467-025-60139-z)
Supplement: Supplementary file 4 — Reporting Summary [file 41467_2025_60139_MOESM4_ESM.pdf]

nature portfolio

Corresponding author(s): Stephen Jackson
Johannes Walter

Last updated by author(s): 04/17/2025

Reporting Summary

Nature Portfolio wishes to improve the reproducibility of the work that we publish. This form provides structure for consistency and transparency in reporting. For further information on Nature Portfolio policies, see our Editorial Policies and the Editorial Policy Checklist.

Statistics

For all statistical analyses, confirm that the following items are present in the figure legend, table legend, main text, or Methods section.

n/a	Confirmed	
☐	☒	The exact sample size (*n*) for each experimental group/condition, given as a discrete number and unit of measurement
☐	☒	A statement on whether measurements were taken from distinct samples or whether the same sample was measured repeatedly
☐	☒	The statistical test(s) used AND whether they are one- or two-sided<br>*Only common tests should be described solely by name; describe more complex techniques in the Methods section.*
☒	☐	A description of all covariates tested
☐	☒	A description of any assumptions or corrections, such as tests of normality and adjustment for multiple comparisons
☐	☒	A full description of the statistical parameters including central tendency (e.g. means) or other basic estimates (e.g. regression coefficient) AND variation (e.g. standard deviation) or associated estimates of uncertainty (e.g. confidence intervals)
☐	☒	For null hypothesis testing, the test statistic (e.g. *F*, *t*, *r*) with confidence intervals, effect sizes, degrees of freedom and *P* value noted<br>*Give P values as exact values whenever suitable.*
☒	☐	For Bayesian analysis, information on the choice of priors and Markov chain Monte Carlo settings
☒	☐	For hierarchical and complex designs, identification of the appropriate level for tests and full reporting of outcomes
☐	☒	Estimates of effect sizes (e.g. Cohen's *d*, Pearson's *r*), indicating how they were calculated

Our web collection on statistics for biologists contains articles on many of the points above.

Software and code

Policy information about availability of computer code

Data collection	Immunoblots were imaged using Amersham Imager 680 or 800 or with ChemiDoc MP 3.0.1.14. DNA fiber images were captured on a Nikon Eclipse Ni microscope.
Data analysis	All structure predictions were generated using AlphaFold multimer v2.3 via ColabFold 1.52<br>AF-M predictions were analysed in UCSF ChimeraX1.8<br>The top ranked models were then relaxed using AMBER (https:// ambermd.org/index.php)<br>Statistical tests were made using R version 4.2.3, Prism v9 or v10.4.1 (GraphPad Software).<br>DNA fiber images were analysed using ImageJ v1.48.<br>Immunoblots in Supplementary Fig. 10 k-l were quantified using band-it tool (https://thecodingbiologist.com/posts/band-it-the-gel-band-quantifier).<br>For PLA data, cells were imaged on a Nikon Eclipse Ni microscope in conjunction with Elements v4.5 software (Nikon)<br>This paper does not report original code.

For manuscripts utilizing custom algorithms or software that are central to the research but not yet described in published literature, software must be made available to editors and reviewers. We strongly encourage code deposition in a community repository (e.g. GitHub). See the Nature Portfolio guidelines for submitting code & software for further information.

Data

Policy information about availability of data

All manuscripts must include a data availability statement. This statement should provide the following information, where applicable:

- Accession codes, unique identifiers, or web links for publicly available datasets
- A description of any restrictions on data availability
- For clinical datasets or third party data, please ensure that the statement adheres to our policy

The authors declare that all data supporting the findings of this study are available within the article and its supplementary information files. Original western blot and gel images are provided in the Source data with this paper. Standardized datasets for Fig. 1a and 2a have been deposited on Dryad (https://datadryad.org/stash/share/uIxGpIs0P4mzIA8Cn9PEecC22u8A0qLK3DawMu2bgns).

Research involving human participants, their data, or biological material

Policy information about studies with human participants or human data. See also policy information about sex, gender (identity/presentation), and sexual orientation and race, ethnicity and racism.

Reporting on sex and gender	N/A
Reporting on race, ethnicity, or other socially relevant groupings	N/A
Population characteristics	N/A
Recruitment	N/A
Ethics oversight	N/A

Note that full information on the approval of the study protocol must also be provided in the manuscript.

Field-specific reporting

Please select the one below that is the best fit for your research. If you are not sure, read the appropriate sections before making your selection.

☒ Life sciences ☐ Behavioural & social sciences ☐ Ecological, evolutionary & environmental sciences

For a reference copy of the document with all sections, see nature.com/documents/nr-reporting-summary-flat.pdf

Life sciences study design

All studies must disclose on these points even when the disclosure is negative.

Sample size	Sample sizes are indicated in the Methods and/or Figure legends. No methods were used to predetermine sample sizes. Instead, sample sizes were chosen based on standards commonly used in the field. Specifically, a minimum of three independent biological replicates for key findings were used, which is widely accepted in life and biological sciences research, which is also a sufficient number of biological replicates to ensure reproducibility and statistical power for detecting meaningful differences.<br>In experiments involving immunoblotting, the chosen sample sizes (at least 3 independent replicates for key findings) were sufficient to observe consistent and reproducible results.
Data exclusions	Outliers in Fig. 3d were removed with the ROUT method using Prism.No other data were excluded from the analysis.
Replication	All attempts of replication were successful. Experiments in Xenopus egg extracts were performed independently three times with the exception of experiments in Extended Data Fig 5b. and 9c, which were performed twice as they are a confirmation of previously published results (Heintzman et al., 2019 Cell Rep; Sparks et al., 2019 Cell; Larsen et al 2019 Mol Cell).
Randomization	This study did not involve allocations into experimental groups and randomization was not relevant.
Blinding	This study did not involve allocations into experimental groups and blinding was not relevant.

Reporting for specific materials, systems and methods

We require information from authors about some types of materials, experimental systems and methods used in many studies. Here, indicate whether each material, system or method listed is relevant to your study. If you are not sure if a list item applies to your research, read the appropriate section before selecting a response.

Materials & experimental systems

n/a	Involved in the study	
☐	☒	Antibodies
☐	☒	Eukaryotic cell lines
☒	☐	Palaeontology and archaeology
☐	☒	Animals and other organisms
☒	☐	Clinical data
☒	☐	Dual use research of concern
☒	☐	Plants

Methods

n/a	Involved in the study	
☒	☐	ChIP-seq
☒	☐	Flow cytometry
☒	☐	MRI-based neuroimaging

Antibodies

Antibodies used

All antibodies are available upon request from the authors or from commercial suppliers.
USP37 Abcam ab72199; 1:1000
mCherry Abcam ab167453; 1:1000
MCM2 Abcam ab4461; 1:1000
MCM6 Bethyl A300-127A; 1:1000
MCM7 Santa Cruz sc-9966; 1:1000
CDC45 Santa Cruz sc-20685; 1:1000
GAPDH Millipore MAB374; 1:1000
TUBULIN Sigma- Aldrich T9026; 1:1000
Biotin Jackson Immunoresearch, 200-002-211; 1:2000
Flag Sigma Aldrich F7425 (1:3000)
CDC45 (Cell Signaling Tech #11881, 1:500)
RPA Calbiochem NA18; 1:1000
γH2AX Cell signaling technologies 2577; 1:1000
MCM7 J. Walter laboratory (Walter & Newport 2000) Pocono Rabbit Farm and Laboratory Custom Project 456; 1:12000
MCM4 Bethyl Laboratories Cat# A300-193A-1; 1:2000
MCM6 J. Walter laboratory (Dewar et al. 2017) New England Peptide Custom Project 2926; 1:5000)
CDC45 J. Walter laboratory (Mimura 1998) Pocono Rabbit Farm and Laboratory Custom Project 534; 1:20000
RPA J. Walter laboratory (Walter & Newport 2000) Pocono Rabbit Farm and Laboratory Custom Project 519; 1:7000
TRAIP J. Walter laboratory (Wu et al. 2019) New England Peptide Custom Project 3472; 1:10000
TOP2a J. Walter laboratory (Dewar et al. 2015) Bethyl Laboratories Custom Project 61587A; 1:5000
USP37.L This study BioSynth Custom Project 5603; 1:5000
SMC2 J. Walter laboratory (Deng et al. 2019) Bethyl Laboratories Custom Project; 1:5000
SMC3 J. Walter laboratory (Takahashi et al. 2004) New England Peptide Custom Project 3532; 1:5000
UBXN7 J. Walter laboratory (Kochenova et al., 2022) Bethyl Laboratories Custom Project; 1:5000
H3 Cell Signaling Cat# 9715S; RRID: AB_331563; 1:500
Ubiquitin Santa Cruz Biotechnology Cat#sc-8017 P4D1; 1:300
RTEL1-N J. Walter laboratory (Sparks et al. 2019) Pocono Rabbit Farm and Laboratory Custom Project 32259; 1:2500
P-H3 (S10) (1:1,000; Cell Signaling, 9701S)
Peroxidase IgG Fraction Monoclonal Mouse Anti-Rabbit IgG, Light Chain Specific Jackson ImmunoResearch Cat#211-032-171; 1:2000
Peroxidase AffiniPure Rabbit Anti-Mouse IgG (H+L) Jackson ImmunoResearch Cat# 315-035-003; 1:10000
Peroxidase-AffiniPure Goat Anti-Rabbit IgG (H+L) Jackson ImmunoResearch Cat# 111-035-003; RRID: AB_2313567; 1:20000

Validation

USP37 Abcam ab72199 was validated by the manufacturer for IP, WB, IHC-P and reacts with Human samples.
mCherry Abcam ab167453 was validated by the manufacturer for WB, ICC/IF and reacts with Tag samples
MCM2 Abcam ab4461 was validated by the manufacturer for IHC-P, IP, WB and reacts with Human, Mouse samples.
MCM6 Bethyl A300-127A was validated by the manufacturer for IHC and WB
MCM7 Santa Cruz sc-9966 was validated by the manufacturer for detection of MCM7 of mouse, rat and human origin by WB, IP, IF, IHC(P) and ELISA
CDC45 Santa Cruz sc-20685 was validated by the manufacturer for WB
GAPDH Millipore MAB374 was validated by the manufacturer for ELISA, IHC, IF, IP, WB.
TUBULIN Sigma- Aldrich T9026 was validated by the manufacturer for indirect IF and WB
Biotin Jackson Immunoresearch, 200-002-211 was validated by the manufacturer RRID:AB_2339006
Flag Sigma Aldrich F7425 was validated by the manufacturer for dot blot, IP,
indirect IF, WB
CDC45 (Cell Signaling Tech #11881) was validated by manufacturer for WB, IP, IF.
RPA Calbiochem NA18 was validated by the manufacturer for WB, IF, IP, paraffin sections.
γH2AX Cell signaling technologies 2577 was validated by the manufacturer for WB, IF, F
All custom antibodies against Xenopus proteins (MCM6, GINS, MCM7, CDC45, TRAIP, USP37.L, SMC3, TOP2a, SMC2, RPA, UBXN7) were validated by Western blotting of the respective antigen and the detection of the band of the expected size by Western blotting Xenopus egg extracts .
H3 Cell Signaling Cat# 9715S is validated for WB, IP, ChIP, IP, IF by manufacturer.
Ubiquitin Santa Cruz Biotechnology Cat# sc-8017 P4D1 is validated by WB. IHC, IF by manufacturer.
P-H3 (S10) Cell Signaling, 9701S is validated by the manufacturer for WB, IHC, IF
Peroxidase IgG Fraction Monoclonal Mouse Anti-Rabbit IgG, Light Chain Specific was validated by the manufacturer and based on antigen-binding assay, Western blotting, and/or ELISA, the antibody reacts with the light chains on rabbit IgG and with those common to other rabbit immunoglobulins. The antibody does not react with the heavy chain of rabbit IgG.

	Peroxidase AffiniPure™ Rabbit Anti-Mouse IgG (H+L) was validated by the manufacturer and based on immunoelectrophoresis and/or ELISA, the antibody reacts with whole molecule mouse IgG.<br>Peroxidase AffiniPure™ Goat Anti-Rabbit IgG (H+L) was validated by the manufacturer and based on immunoelectrophoresis and/or ELISA, the antibody reacts with whole molecule rabbit IgG.<br>MCM4 Bethyl Laboratories Cat# A300-193A-1 antybodies were validated by the manufacturer for WB, IHC, IP.

Eukaryotic cell lines

Policy information about cell lines and Sex and Gender in Research

Cell line source(s)	RPE-1 and HEK293T cells were originally obtained from Prof. Jonathon Pines. RPE-1 TP53KO were previously reported in Chiang, 2016. U2OS Cas9 were previously reported in Lloyd, 2021. HEK293T Lenti-X were obtained from Takara Bio.
Authentication	RPE-1 and U2OS were authenticated via STR profiling
Mycoplasma contamination	All cell lines tested negative for mycoplasma contamination
Commonly misidentified lines (See ICLAC register)	Not applicable

Animals and other research organisms

Policy information about studies involving animals; ARRIVE guidelines recommended for reporting animal research, and Sex and Gender in Research

Laboratory animals	Xenopus laevis were purchased from Nasco Cat #LM0053MX. Females used for egg collection were aged >2 years.
Wild animals	This study did not involve wild animals.
Reporting on sex	This study did not involve sex-based analysis.
Field-collected samples	This study did not involve field-collected samples.
Ethics oversight	All experiments involving animals were approved by the Harvard Medical Area Standing Committee on Animals (HMA IACUC Study ID IS00000051-6, approved 10/23/2020, and IS00000051-9, approved 10/23/2023). The Harvard Medical School has an approved Animal Welfare Assurance (D16-00270) from the NIH Office of Laboratory Animal Welfare.

Note that full information on the approval of the study protocol must also be provided in the manuscript.

Plants

Seed stocks	N/A
Novel plant genotypes	N/A
Authentication	N/A
